# Loss of intratumoral macroscopic fat in renal angiomyolipoma following chemoradiation therapy for pancreatic cancer

**DOI:** 10.1259/bjrcr.20150439

**Published:** 2016-10-14

**Authors:** Frank Howard Miller, Jeet Minocha, Sudharshan Parthasarathy, Sharon Zahava Adam, Carolina Parada, Vahid Yaghmai

**Affiliations:** ^1^Department of Radiology, Northwestern Memorial Hospital, Northwestern University, Feinberg School of Medicine, Evanston, IL, USA; ^2^Department of Radiology, University of California, San Diego, CA, USA

## Abstract

Angiomyolipoma (AML) is the most common benign mesenchymal tumour of the kidney. Classically, AML can readily be diagnosed by identifying the negatively attenuating intratumoral macroscopic fat component on non-enhanced CT scans. However, intratumoral macroscopic fat may not be visible on CT scans, mimicking renal cell carcinoma. We report a case of renal AML with CT scan evidence of macroscopic intratumoral fat that was not readily visible on subsequent CT or MRI, presumably owing to a generalized rapid loss of adipose tissue due to cachexia in a patient with pancreatic adenocarcinoma. Radiologists should be aware that AML may lose its intratumoral fat on follow-up imaging and may simulate renal cell carcinoma.

## Case report

A 64-year-old female with newly diagnosed Stage 1B pancreatic adenocarcinoma was treated by neoadjuvant gemcitabine chemotherapy and radiation, followed by a Whipple procedure at an outside institution 1 month after the completion of neoadjuvant therapy, resulting in a substantial weight loss of 40 pounds (18  kg). The patient was admitted to our institution with dizziness and hypotension.

**Figure 1.**
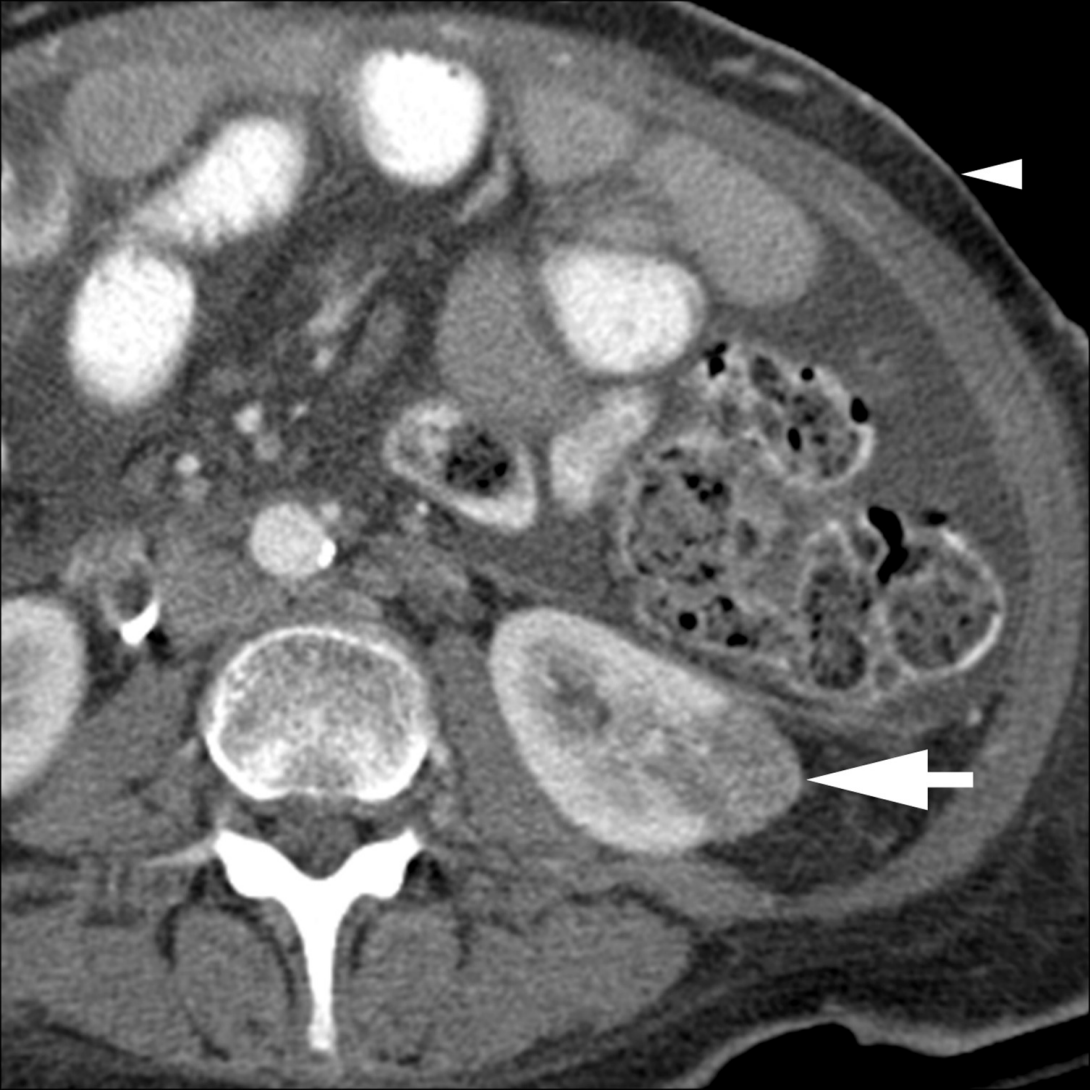
Contrast-enhanced CT scan of the abdomen and pelvis following treatment for pancreatic adenocarcinoma showing an enhancing lesion at the lower pole of the left kidney measuring 2.5 cm (large arrow). Evidence of macroscopic fat suggesting angiomyolipoma is not readily identified, likely owing to significant loss of fat (small arrowhead).

**Figure 3.**
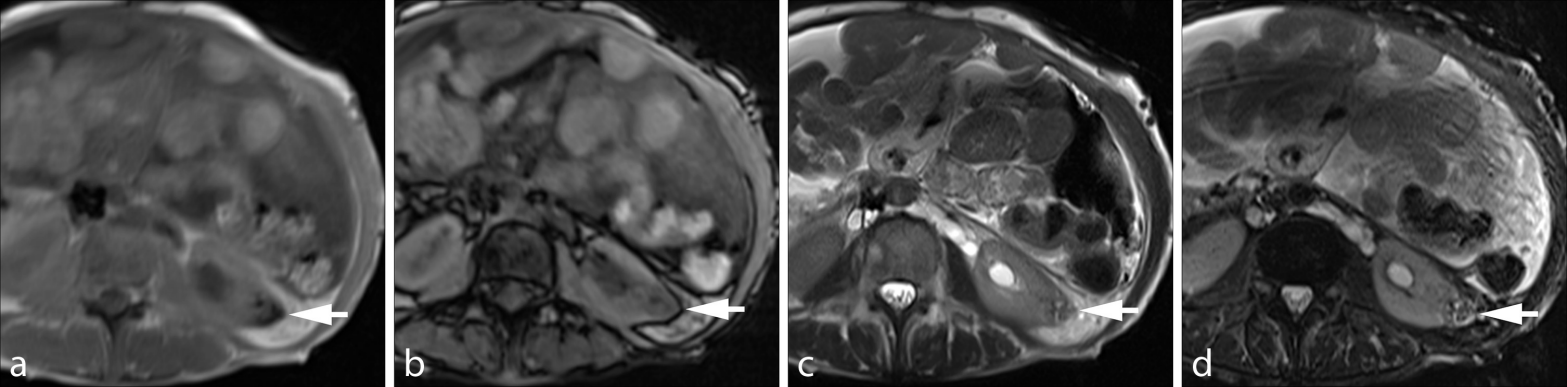
An MR examination more than 2 years after the initial CT scan shows the angiomyolipoma (arrows) with lack of microscopic intratumoral fat on the in-phase (a) and opposed-phase images (b). A small focus of low signal intensity seen on both images may relate to haemosiderin. In addition, *T*_2_ weighted images without (c) and with fat suppression (d) demonstrate the lack of macroscopic intratumoral fat.

**Figure 4.**
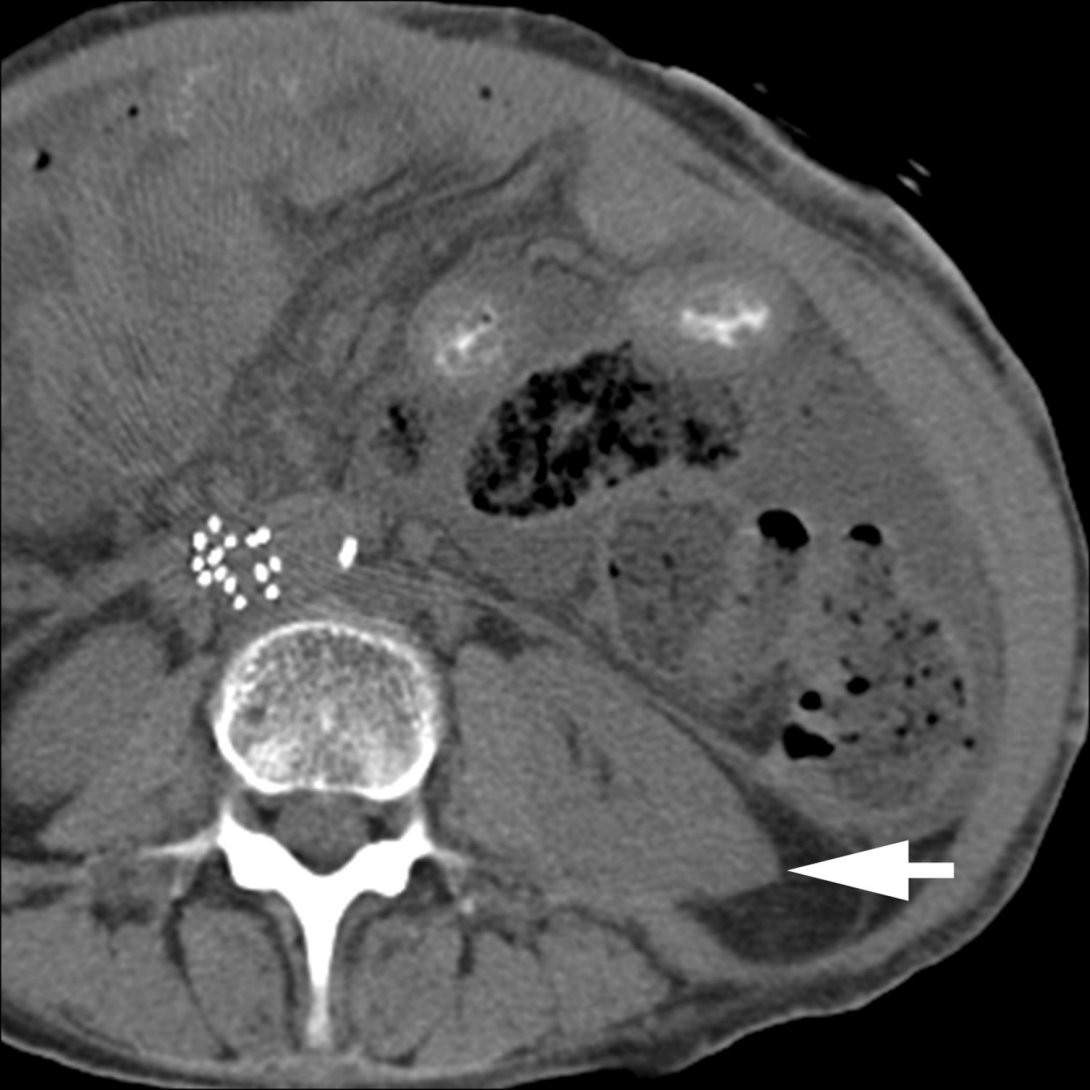
An unenhanced CT scan performed almost 2 years after the initial CT scan shows that the mass has homogeneous attenuation (arrow), with minimal hyperattenuation of the lesion (35 Hounsfield units) compared with the surrounding renal parenchyma (32 Hounsfield units), with attenuation ratio of 1.09.

A contrast-enhanced CT scan of the abdomen was performed with a 16-slice multidetector CT scanner (Somatom Sensation 16, Siemens Medical Solutions, Forchheim, Germany) using a routine single-phase abdomen and pelvis protocol in the portal venous phase after administration of 125  ml of iohexol 350 mg ml^−1^ (Omnipaque 350, GE Healthcare, Milwaukee, WI) at a rate of 3 ml s^−1^ with 5 mm slice thickness and 120 kV. The contrast-enhanced CT scan revealed an incidental solid enhancing mass measuring 2.5 cm in diameter in the lower pole of the left kidney, which was suspicious for renal cell carcinoma (RCC; Figure 1). On comparison with pre-treatment imaging performed 9 months earlier, the mass corresponded to a 3.0 cm lesion (Figure 2) that demonstrated regions of negative attenuation (−39 Hounsfield units), characteristic of intratumoral fat and consistent with angiomyolipoma (AML). The subcutaneous fat thickness seen on the post-chemotherapy CT scan was 1.0 cm, compared with 2.5 cm on the pre-treatment CT scan, and consistent with the substantial reported weight loss. Several CT and MR examinations of the abdomen were subsequently performed for various reasons unrelated to the AML, demonstrating continued decrease in the size of the mass, reaching a size of 2.0 cm. The intratumoral fat component remained imperceptible on imaging, including an MR of the abdomen performed more than 2 years after the initial CT (Figure 3) and an unenhanced CT that had shown minimal hyperattenuation of the mass compared with the surrounding parenchyma (Figure 4).

**a:**
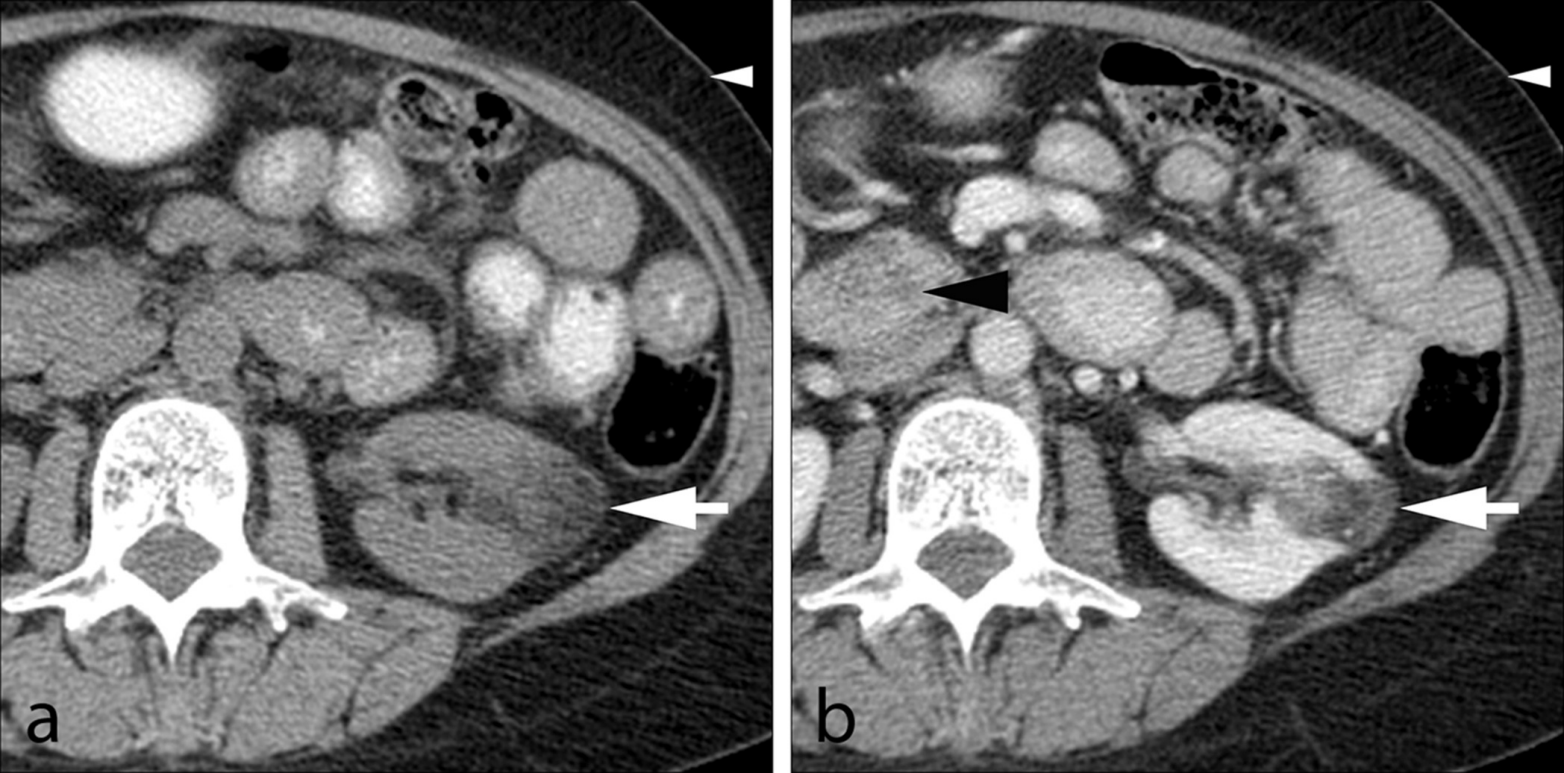
() Unenhanced and (b) contrast-enhanced CT scans of the abdomen and pelvis before treatment for pancreatic adenocarcinoma shows an incidental 3.0 cm lesion at the lower pole of the left kidney with areas of fat attenuation (large white arrows), consistent with angiomyolipoma. The contrast-enhanced CT scan shows the pancreatic head mass from adenocarcinoma (black arrowhead). A noteworthy feature is the abundance of subcutaneous fat compared with imaging done after treatment (small white arrowheads).

## Discussion

Although it constitutes only 1–2% of all tumours seen in the kidney, renal AML is the most common benign renal neoplasm, comprising variable amounts of abnormal blood vessels, smooth muscle and adipose tissue.^1^ While AML most commonly occurs sporadically, up to 20% can occur in association with the tuberous sclerosis complex.^2^ AML is typically diagnosed incidentally in patients undergoing imaging work-up for other diseases.^3^ Demonstration of negatively attenuating intratumoral fat on unenhanced CT scans usually provides pathognomonic evidence of AML and virtually excludes the diagnosis of RCC,^4^ although fat-containing RCCs have been rarely described when the RCC engulfs normal adjacent renal sinus or perinephric fat, when osseous metaplasia results in marrow content without ossification or when cholesterol necrosis mimics the presence of true adipose tissue.^5^ Other rare renal tumours may also contain fat and mimic the appearance of AMLs, such as Wilms tumours, liposarcomas, oncocytomas and solitary fibrous tumours.^4,6^ However, in rare cases, the tumour may be composed mostly of smooth muscle and blood vessels, with only a minimal amount of mature adipose tissue that is not detected on CT scans. These minimal fat renal AMLs contain only 3–10% mature adipose tissue^^2,7^^ and pose a diagnostic dilemma for even experienced radiologists.^8^ Minimal fat renal AML and RCC, mostly clear cell and papillary RCCs, but also the more rare chromophobe and sarcomatoid RCCs, can have similar imaging appearances on CT scans, particularly when small in size (< 4 cm).^9^ Since small renal AMLs measuring < 4 cm have traditionally been managed conservatively based on their lack of malignancy and decreased propensity for haemorrhage, and masses > 4 cm may undergo selective embolization rather than partial or radical nephrectomy, distinguishing renal AML from RCC is critical in clinical management.^10^

Several different imaging parameters and techniques have been investigated to distinguish minimal fat renal AML from RCC using CT and MR, but none have proven conclusive thus far.^11^ On unenhanced CT scans, minimal fat AMLs have typically been described as homogeneous and hyperattenuating when compared with surrounding renal parenchyma,^12,13^ with tumour-to-cortex attenuation ratio significantly higher for minimal fat AML compared with clear cell and papillary RCCs, but not significantly different from the ratio in other types of RCC.^7^ This lack of specificity precluded this finding from being used as a reliable differentiating factor between benign and malignant tumours. Homogeneity of tumour enhancement and a prolonged enhancement pattern are the most valuable CT findings for differentiating minimal fat AML from RCC,^12^ but are also not reliable enough. CT attenuation histogram analysis using pixel distribution analysis and counts of negative attenuation pixels has shown mixed results.^14–16^ CT texture analysis has shown promise in differentiating minimal fat AML from RCC^17^ but has not been studied sufficiently. Zhang et al^18^ used CT parameters such as unenhanced attenuation characteristic, intratumoral vessels, and attenuation values of unenhanced and early excretory phase scans to differentiate minimal fat AML from papillary RCC. While other reports have shown that CT and MR can aid in distinguishing clear cell RCC from minimal fat AML,^14,19^ Hindman et al^11^ have shown that differentiation between minimal fat AML and clear cell RCC often is not possible on the basis of MR alone, and others have shown that the MR appearance of minimal fat AML and papillary RCC overlap.^7^

We present a case of renal AML in which CT evidence of intratumoral macroscopic fat disappeared on subsequent imaging, presumably owing to rapid interval loss of large amounts of adipose tissue due to cancer cachexia. A serious side effect of malignancy occurring in nearly half of cancer patients, cachexia is a multifactorial syndrome characterized by depletion of > 10% body weight, loss of adipose tissue and skeletal muscle mass, and alterations in metabolic homeostasis.^20^ While the mechanisms for muscle degradation in cancer cachexia have been well studied,^21^ the mechanisms behind adipose tissue loss are only recently being elucidated. Activation of the acute phase response cascade^22^ and enhanced expression and function of adipocyte hormone-sensitive lipase^23^ have recently been proposed as the primary mediators of fat loss in cancer cachexia. Combined with an elevated resting energy expenditure and lower oral food intake,^24^ pancreatic cancer cachexia produces a dramatic reduction in body fat with a nearly twofold increase in lipolytic activity mediated by hormone-sensitive lipase.^23^ The patient’s significant weight loss and loss of measurable adipose tissue are readily identified on CT scans prior to and following neoadjuvant chemoradiation therapy (Figures 1 and 2).

This case also illustrates the importance of comparison with previous imaging studies before planning for intervention in a solid renal mass. Although the morphology of solid renal masses may vary over time with changes in enhancement pattern or development of regions of necrosis, loss of macroscopic fat in a solid renal tumour has not been reported to our knowledge. Review of prior imaging studies in patients with solid renal masses concerning for RCC, especially when patients have a known malignancy or report marked weight loss, may help detect prior presence of intratumoral macroscopic fat to avoid misdiagnosing AML as RCC.

## Learning points

1. Renal masses that lack fat may nonetheless be AMLs.
2. The loss of macroscopic fat in a solid renal tumour may be caused by rapid weight loss related to cancer or chemoradiation therapy.
3. It is important to review old scans, if available, to assess for the presence of intratumoral macroscopic fat to avoid misdiagnosis of AML as RCC.

## Consent

This paper has been sufficiently anonymized to protect patient identify. Despite multiple attempts, informed consent could not be obtained.

## References

[1] Lienert AR, Nicol D. Renal angiomyolipoma. BJU Int 2012; 110 Suppl 4: 25–7. 23194120 10.1111/j.1464-410X.2012.11618.x

[2] Hajdu SI, Foote FW. Angiomyolipoma of the kidney: report of 27 cases and review of the literature. J Urol 1969; 102: 396–401. 5343474 10.1016/s0022-5347(17)62157-8

[3] Halpenny D, Snow A, McNeill G, Torreggiani WC. The radiological diagnosis and treatment of renal angiomyolipoma-current status. Clin Radiol 2010; 65: 99–108. 20103431 10.1016/j.crad.2009.09.014

[4] Hélénon O, Merran S, Paraf F, Melki P, Correas JM, Chrétien Y, et al Unusual fat-containing tumors of the kidney: a diagnostic dilemma. Radiographics 1997; 17: 129–44. 9017804 10.1148/radiographics.17.1.9017804

[5] Richmond L, Atri M, Sherman C, Sharir S. Renal cell carcinoma containing macroscopic fat on CT mimics an angiomyolipoma due to bone metaplasia without macroscopic calcification. Br J Radiol 2010; 83: e179–81. 20647509 10.1259/bjr/46452134 PMC3473503

[6] Chen Y, Wang F, Han A. Fat-forming solitary fibrous tumor of the kidney: a case report and literature review. Int J Clin Exp Pathol 2015; 8: 8632–5. 26339447 PMC4555775

[7] Jeong CJ, Park BK, Park JJ, Kim CK. Unenhanced CT and MRI parameters that can be used to reliably predict fat-invisible angiomyolipoma. AJR Am J Roentgenol 2016; 206: 340–7. 26797361 10.2214/AJR.15.15086

[8] Silverman SG, Israel GM, Herts BR, Richie JP Management of the incidental renal mass. Radiology 2008; 249: 16–31. 18796665 10.1148/radiol.2491070783

[9] Chaudhry HS, Davenport MS, Nieman CM, Ho LM, Neville AM. Histogram analysis of small solid renal masses: differentiating minimal fat angiomyolipoma from renal cell carcinoma. AJR Am J Roentgenol 2012; 198: 377–83. 22268181 10.2214/AJR.11.6887

[10] Nelson CP, Sanda MG. Contemporary diagnosis and management of renal angiomyolipoma. J Urol 2002; 168: 1315–25. 4. 12352384 10.1016/S0022-5347(05)64440-0

[11] Hindman N, Ngo L, Genega EM, Melamed J, Wei J, Braza JM, et al Angiomyolipoma with minimal fat: can it be differentiated from clear cell renal cell carcinoma by using standard MR techniques? Radiology 2012; 265: 468–77. 23012463 10.1148/radiol.12112087 PMC3480813

[12] Kim JK, Park SY, Shon JH, Cho KS. Angiomyolipoma with minimal fat: differentiation from renal cell carcinoma at biphasic helical CT. Radiology 2004; 230: 677–84. 14990834 10.1148/radiol.2303030003

[13] Silverman SG, Mortele KJ, Tuncali K, Jinzaki M, Cibas ES Hyperattenuating renal masses: etiologies, pathogenesis, and imaging evaluation. Radiographics 2007; 27: 1131–43. 17620471 10.1148/rg.274065147

[14] Catalano OA, Samir AE, Sahani DV, Hahn PF Pixel distribution analysis: can it be used to distinguish clear cell carcinomas from angiomyolipomas with minimal fat? Radiology 2008; 247: 738–46. 18413886 10.1148/radiol.2473070785

[15] Simpfendorfer C, Herts BR, Motta-Ramirez GA, Lockwood DS, Zhou M, Leiber M, et al Angiomyolipoma with minimal fat on MDCT: can counts of negative-attenuation pixels aid diagnosis? AJR Am J Roentgenol 2009; 192: 438–43. 19155407 10.2214/AJR.08.1180

[16] Kim JY, Kim JK, Kim N, Cho KS. CT histogram analysis: differentiation of angiomyolipoma without visible fat from renal cell carcinoma at CT imaging. Radiology 2008; 246: 472–9. 18094264 10.1148/radiol.2462061312

[17] Yan L, Liu Z, Wang G, Huang Y, Liu Y, Yu Y, et al Angiomyolipoma with minimal fat: differentiation from clear cell renal cell carcinoma and papillary renal cell carcinoma by texture analysis on CT images. Acad Radiol 2015; 22: 1115–21. 26031228 10.1016/j.acra.2015.04.004

[18] Zhang YY, Luo S, Liu Y, Xu RT. Angiomyolipoma with minimal fat: differentiation from papillary renal cell carcinoma by helical CT. Clin Radiol 2013; 68: 365–70. 23321146 10.1016/j.crad.2012.08.028

[19] Kim JK, Kim SH, Jang YJ, Ahn H, Kim CS, Park H, et al Renal angiomyolipoma with minimal fat: differentiation from other neoplasms at double-echo chemical shift FLASH MR imaging. Radiology 2006; 239: 174–80. 16507752 10.1148/radiol.2391050102

[20] Tisdale MJ. Biology of cachexia. J Natl Cancer Inst 1997; 89: 1763–73. 9392617 10.1093/jnci/89.23.1763

[21] Tijerina AJ. The biochemical basis of metabolism in cancer cachexia. Dimens Crit Care Nurs 2004; 23: 237–43. 15586034 10.1097/00003465-200411000-00001

[22] Fearon KC, Barber MD, Falconer JS, McMillan DC, Ross JA, Preston T. Pancreatic cancer as a model: inflammatory mediators, acute-phase response, and cancer cachexia. World J Surg 1999; 23: 584–8. 10227928 10.1007/pl00012351

[23] Agustsson T, Rydén M, Hoffstedt J, van Harmelen V, Dicker A, Laurencikiene J, et al Mechanism of increased lipolysis in cancer cachexia. Cancer Res 2007; 67: 5531–7. 17545636 10.1158/0008-5472.CAN-06-4585

[24] Bosaeus I, Daneryd P, Svanberg E, Lundholm K. Dietary intake and resting energy expenditure in relation to weight loss in unselected cancer patients. Int J Cancer 2001; 93: 380–3. 11433403 10.1002/ijc.1332

